# The Police Department in the Emergency Department: Developing a Patient‐Centered Resource for Navigating Law Enforcement Interactions in Clinical Care Settings

**DOI:** 10.1111/acem.70169

**Published:** 2025-10-11

**Authors:** Emily F. Seeburger, Rucha Alur, Diane N. Haddad, Rodney Babb, Christopher Edwards, Elinore J. Kaufman

**Affiliations:** ^1^ Department of Emergency Medicine, Perelman School of Medicine University of Pennsylvania Philadelphia Pennsylvania USA; ^2^ Center for Health Justice, Center for Healthcare Transformation and Innovation, Penn Medicine Philadelphia Pennsylvania USA; ^3^ Department of Surgery, Perelman School of Medicine University of Pennsylvania Philadelphia Pennsylvania USA; ^4^ Section of Trauma and Acute Care Surgery, Department of Surgery, Pritzker School of Medicine University of Chicago Chicago Illinois USA; ^5^ Division of Traumatology, Surgical Critical Care, and Emergency Surgery, Perelman School of Medicine University of Pennsylvania Philadelphia Pennsylvania USA

Patients presenting for trauma, substance abuse, mental health crisis, or other reasons may encounter law enforcement during an ED visit [1]. Often, law enforcement officers (LEOs) are investigating a potential crime or seeking information, even if the patient is not in custody. These patients are at risk for adverse consequences including stress and compromised privacy. As a result, medical professionals and law enforcement (LE) often converge in the emergency department and in the trauma bay [2, 3].

Little health system, professional, or legal guidance exists to help clinicians, LEOs, and patients navigate these interactions. Clinicians report being confused about what information they are required to disclose about patients to law enforcement and who provides consent. In a study of emergency physicians, only 13% reported that their institution had a policy for these situations, and many had only a vague awareness of what that policy might be [3]. Despite police officer perceptions of the ED as an extension of public space, hospitals and EDs have the ability as well as the right to prevent unrestricted access of LEOs to patient care areas [4]. Like other visitors, law enforcement officers must abide by hospital policies for entry. The absence of clear and enforced policies often allows LEOs largely unrestricted access to patient care areas that can violate patients' rights to privacy. Additionally, patients can refuse to speak with police officers as choosing to speak to the police is always voluntary. However, for patients with an acute health need, exercising their legal rights can be challenging [5].

Patients may also be confused about their legal rights and requirements and have little recourse while they are also seeking medical care. Research has demonstrated that the presence of LEOs in a hospital setting erodes trust between patients and clinicians and compromises care [6]. This has important health equity implications as well. Black patients are significantly more likely to be transported by police to the hospital than White patients, and patients transported by police have five times higher odds of being subsequently restrained at the hospital [7]. Furthermore, Black individuals, and Black men in particular, are disproportionately victims of penetrating trauma and thus are more likely to face law enforcement interaction in the ED [8].

While several toolkits and policies, including two published by the American College of Emergency Physicians, exist for medical professionals on navigating law enforcement interactions and protecting patient privacy in the emergency department, we are not aware of an existing resource for patients that clearly outlines their individual legal rights in a healthcare context [4, 9]. We aimed to create a simple, easy‐to‐read guide that can be distributed to patients coming into the hospital to inform them of their rights, responsibilities, and options when interacting with LEOs.

After reviewing the existing literature on the topic, we consulted legal experts, bioethicists, and emergency department faculty about what legal information would be most relevant for patients. We then created a first version of the patient‐facing resource and presented it to EM faculty for feedback. Based on their feedback, we created a second version of the resource to survey test with patients. Patient input and a second round of ED stakeholder input resulted in the final version of the resource (Figure 1).

**FIGURE 1:**
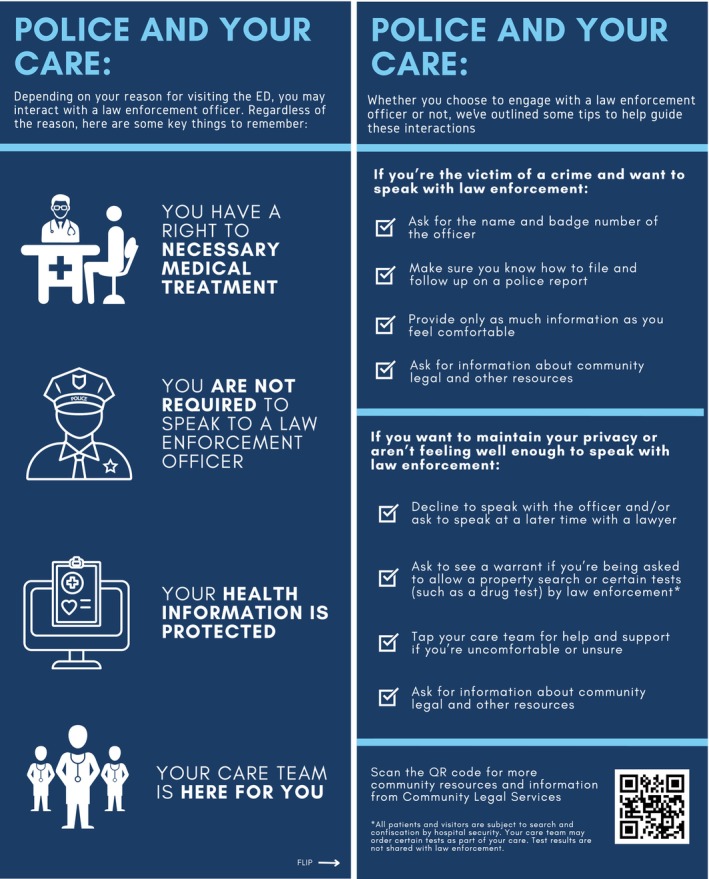
Patient‐facing resource.

This survey study was administered at an urban, academic, level I trauma center emergency department, the inpatient trauma service affiliated with the medical center, and the outpatient surgical clinic for the trauma service. Patients were selected via convenience sampling; all participants were adults who were medically stable and able to consent. Participants were consented verbally. We developed a survey instrument with closed and open‐ended questions to identify what language, information, and design elements are important to patients, as well as how the availability of this information impacts their perspective of the care they received. Participants who had interacted with a LEO during their most recent hospital admission were queried as to their experience. Demographic information, including age, race, ethnicity, and gender, was also collected optionally. The University of Pennsylvania Institutional Review Board approved this study. Written consent was waived since the study presented no more than minimal risk to participants. Surveys were conducted between October 2023 and November 2024.

Survey data were descriptively analyzed to generate frequency counts and percentages for each item. Open‐ended questions were utilized to elicit more detailed feedback on design and content and contributed to our revision process. Of patients surveyed, 17 (89%) were Black, 16 (84%) were men. Median age was 36 (IQR 23.5). We recruited 10 patients in the ED (52.5%), 5 (26%) from the inpatient trauma service, and 4 (21%) from the outpatient trauma surgery clinic. All trauma service patients (*n* = 9) were recovering from a violent injury (gunshot wound or stabbing) and had interacted with a LEO during their most recent hospitalization. None of the emergency department patients we surveyed had encountered a LEO during their current visit.

The majority (17, 89%) of our sample agreed that knowing their legal rights would improve their experience in the hospital, and 15 (79%) reported they would feel cared for if someone on their clinical team shared the resource during their hospital stay. Three (16%) reported that receiving the tool made them feel nervous or uneasy.

Over half of patients (11, 58%) agreed that they liked the design of the resource and that they were able to understand the language describing their legal rights (14, 74%). Only 2 (11%) participants stated they did not like the design, with the rest (6, 32%) reporting that they felt neutral. We received open‐ended feedback that some of the language was too vague and that a design with images and bigger, more straightforward text would be useful. One patient also suggested creating a digital version that could be accessed via QR code.

Of the nine trauma patients who interacted with LEO during their hospital stay, 6 of 9 (67%) reported that the interaction negatively affected their hospital visit. Patients reported being questioned by a LEO soon after receiving pain medication in the trauma bay, and as a result felt more vulnerable during questioning, with one patient reporting he did not remember what he said to a LEO. Participants also reported frustration that there was no follow‐up from police after initial questioning (“I want information on my case and the detective won't call me back.” “I want to see the video of me getting shot for closure, but they [LE] won't get back to me.”).

This study has several limitations. This was a small pilot study at a single institution, and other settings may yield other results. We have yet to deploy the resource in clinical care and look forward to studying its utility in practice. We hope that it will also be adaptable to other settings.

In the absence of clear policy, clinician‐LEO‐patient interactions are often managed ad hoc, leading to confusion, conflict, and at times impeding or impairing patient‐centered care. Along with developing institutional policies and educating clinicians, providing patients with basic information on their role and rights can help them feel cared for and informed. LEO contact can impair patient trust in the clinical team, and a resource like this can open a dialog that may foster greater trust. Such resources may be increasingly relevant if immigration enforcement becomes more common in hospitals, as promised by the current administration [10].

## Author Contributions

E.F.S. and E.J.K. were involved in study concept and design. E.F.S., R.A., R.B., and D.N.H. were involved in acquisition of the data. E.F.S. and E.J.K. were involved in analysis and interpretation of the data. E.F.S. was involved in drafting of the manuscript. E.F.S., R.A., D.N.H., R.B., C.E., and E.J.K. were involved in critical revision of the manuscript for important intellectual content. E.F.S. was involved in statistical expertise. E.F.S. and E.J.K. were involved in acquisition of funding.

## Conflicts of Interest

The authors declare no conflicts of interest.

## Data Availability

The data that support the findings of this study are available on request from the corresponding author. The data are not publicly available due to privacy or ethical restrictions.

## References

[1] M. R. Tahouni , E. Liscord , and H. Mowafi , “Managing Law Enforcement Presence in the Emergency Department: Highlighting the Need for New Policy Recommendations,” Journal of Emergency Medicine 49, no. 4 (2015): 523–529, 10.1016/j.jemermed.2015.04.001. 26095221

[2] E. J. Kaufman , U. G. Khatri , E. C. Hall , R. Alur , J. Song , and S. F. Jacoby , “Law Enforcement in the Trauma Bay: A Survey of Members of the American Academy for the Surgery of Trauma,” Trauma Surgery & Acute Care Open 8 (2023): e001022. 36937171 10.1136/tsaco-2022-001022 PMC10016311

[3] U. G. Khatri , E. J. Kaufman , E. F. Seeburger , et al., “Emergency Physician Observations and Attitudes on Law Enforcement Activities in the Emergency Department,” Western Journal of Emergency Medicine 24, no. 1 (2023): 160–168. 36976602 10.5811/westjem.2022.12.57098 PMC10047729

[4] Working Group on Policing and Patient Rights , “Police in the Emergency Department: A Medical Provider Toolkit for Protecting Patient Privacy,” (2021), https://www.law.georgetown.edu/health‐justice‐alliance/wp‐content/uploads/sites/16/2021/05/Police‐in‐the‐ED‐Medical‐Provider‐Toolkit.pdf.

[5] J. S. Song , “Policing the Emergency Room,” Harvard Law Review 134 (2020): 2646.

[6] K. Gallen , J. Sonnenberg , C. Loughran , et al., “Health Effects of Policing in Hospitals: A Narrative Review,” Journal of Racial and Ethnic Health Disparities 10, no. 2 (2023): 870–882, 10.1007/s40615-022-01275-w. 35267188

[7] M. W. Wandling , A. B. Nathens , M. B. Shapiro , and E. R. Haut , “Police Transport Versus Ground EMS: A Trauma System‐Level Evaluation of Prehospital Care Policies and Their Effect on Clinical Outcomes,” Journal of Trauma and Acute Care Surgery 81, no. 5 (2016): 931–935, 10.1097/TA.0000000000001228. 27537514

[8] J. A. Bailey , S. F. Jacoby , E. C. Hall , U. Khatri , G. Whitehorn , and E. J. Kaufman , “Compounding Trauma: The Intersections of Racism, Law Enforcement, and Injury,” Current Trauma Reports 8, no. 3 (2022): 105–112, 10.1007/s40719-022-00231-7. 35578594 PMC9096065

[9] American College of Emergency Physicians , “Law Enforcement Presence in the Emergency Department: A Toolkit by the State Legislative & Regulatory Committee Developed in Collaboration With the Diversity, Equity, & Inclusion Committee,” (2023), https://www.acep.org/state‐advocacy/state‐advocacy‐overview/law‐enforcement‐toolkit.

[10] American College of Emergency Physicians , “Navigating Immigration Enforcement in the Emergency Department,” (2025), https://www.acep.org/siteassets/new‐pdfs/advocacy/navigating‐immigration‐enforcement‐in‐the‐ed.pdf.

